# Supplementary material for: The number of active metabolic pathways is bounded by the number of cellular constraints at maximal metabolic rates
Source: PLoS Comput Biol. 2019 Mar 11;15(3):e1006858. doi: 10.1371/journal.pcbi.1006858 (PMC6428345; doi:10.1371/journal.pcbi.1006858)
Supplement: S2 Appendix — In this short text we analyse a core model of overflow metabolism using the extremum principle. Code for running the model is also provided. (PDF) [file pcbi.1006858.s002.pdf]

S2 Appendix: Core model of overflow metabolism

Daan H. de Groot, Coco van Boxtel, Robert Planqué, Frank J. Bruggeman, Bas Teusink
January 15, 2019

The core model of overflow metabolism which was referred to in the main text was made using Matlab (Matlab-files are supplemented). In this model we picked an objective reaction: the reaction towards biomass. The flux through the objective reaction was fixed and we minimized the substrate concentration, which is analogous to the actual selective pressure in a chemostat. The Matlab programs (which are attached to the SI) return the optimal concentrations of all metabolites and enzymes.

The cost vectors that were shown in the figures in the main text were calculated at the optimal metabolite concentrations. We calculated the necessary fractions of the constrained enzyme pools to obtain one unit of enzyme flux, according to the definition of a cost vector given in the main text and in S1 Appendix. The alternative positions of the cost vectors are obtained by starting from the optimal concentrations and then vary one of the intracellular metabolite concentrations.

The rate equations used were all of the form $v_i = k_{\text{cat},i} e_i f_i(\boldsymbol{x})$. In the overflow metabolism model we used a transporter (v_{tr}) reaction, a respiration (v_r) and a fermentation (v_f) reaction and a biomass v_{BM} reaction. More detailed results of this model can be found in 1.

A remarkable aspect of this simulation is that the flux through respiration is before the critical growth rate not proportional to the concentration of respiration proteins. This behaviour is found in various experiments [1,2] and cannot be explained by current models that fix enzyme saturations. In fact, the presence of two separate enzyme constraints causes this behaviour: the respiration proteins (which are part of the first constrained pool) are expressed at low growth rates to reduce the cost of the EFM for the transporter pool by reducing product inhibition by keeping internal glucose levels low.

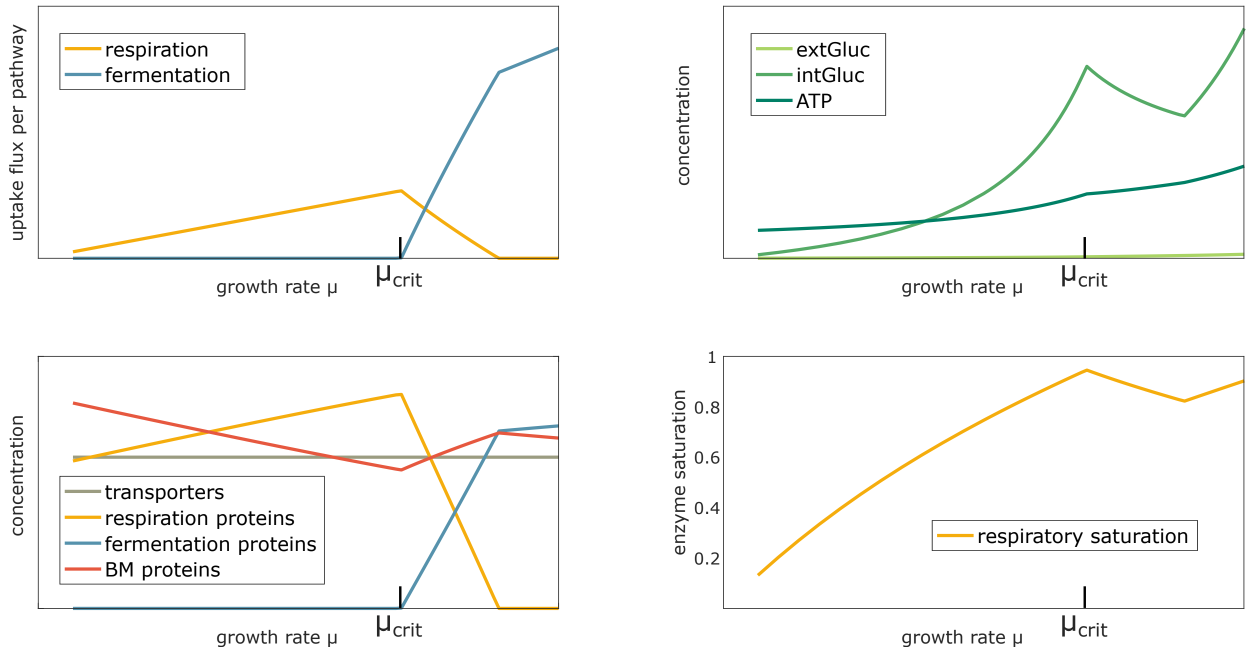

Figure 1: Additional model results from our core model of overflow metabolism. The flux through respiration increases proportionally to the growth rate until the critical growth rate μ_{crit}, after which respiratory flux decreases and fermentation flux increases. In our model, the membrane constraint is always saturated, indicated by a constant level of transporters. Respiratory proteins do increase slightly with growth rate, but not with the same factor as respiratory flux, indicating an increase in their activity: indeed, internal glucose levels increase up to the critical growth rate.

0.1 Source code

Code for running kinetic model of overflow metabolism The Matlab-code used for modeling overflow metabolism is attached in a compressed folder as a supplement. In the compressed folder, we have also added a text-file with instructions.

References

[1] Goel A, Eckhardt TH, Puri P, de Jong A, dos Santos F, Giera M, et al. Protein costs do not explain evolution of metabolic strategies and regulation of ribosomal content: does protein investment explain an anaerobic bacterial Crabtree effect? Molecular Microbiology. 2015;97(1):77–92.

[2] Canelas AB, Ras C, ten Pierick A, van Gulik WM, Heijnen JJ. An in vivo data-driven framework for classification and quantification of enzyme kinetics and determination of apparent thermodynamic data. Metabolic Engineering. 2011;13(3):294–306.
