# Supplementary material for: The number of active metabolic pathways is bounded by the number of cellular constraints at maximal metabolic rates
Source: PLoS Comput Biol. 2019 Mar 11;15(3):e1006858. doi: 10.1371/journal.pcbi.1006858 (PMC6428345; doi:10.1371/journal.pcbi.1006858)
Supplement: S3 Appendix — In this short text we analyse a core model of overflow metabolism in L. lactis using the extremum principle. Code for running the model is also provided. (PDF) [file pcbi.1006858.s003.pdf]

S3 Appendix: Core model of *L. lactis* switch

Daan H. de Groot, Coco van Boxtel, Robert Planqué, Frank J. Bruggeman, Bas Teusink
January 15, 2019

The core model of the metabolic switch by *L. lactis* which was referred to in the main text was made using Matlab (Matlab-files are supplemented). In this model we picked the reaction towards biomass as the objective function again. This objective function is maximized under enzyme constraints (a membrane and a cytosolic constraint), and given glucose and pyruvate concentrations. Here, we make the assumption that the pyruvate concentration is not only determined by glycolysis, but also by other cellular processes. To model this more accurately, these cellular processes should be included, but this is beyond the scope of the paper. This model was merely made to emphasize that protein concentrations can remain constant while pathway usage changes. The Matlab programs (which are attached to the SI) return the optimal concentrations of ATP and all enzymes, and an estimated growth rate.

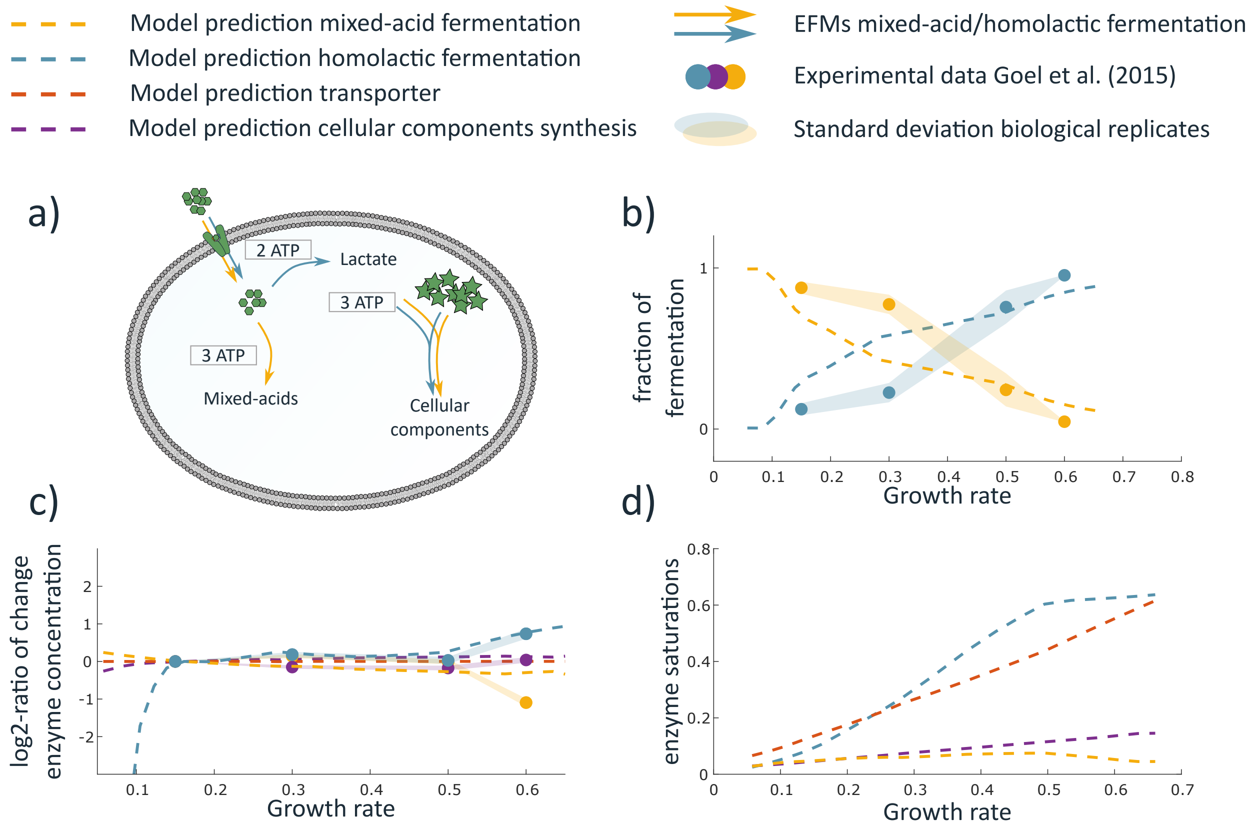

Figure 1: **a)** Core model of energy-limited *L. lactis*-metabolism. ATP can be generated with mixed-acid fermentation (yielding a total of 3 ATP/glucose) and homolactic fermentation (2ATP/glucose). We assume that the cell synthesis reaction is limited only by ATP-concentration. **b-c)** Model predictions and data of the fermentation fluxes and enzyme concentrations. **d)** The change of enzyme saturations as a function of growth rate. The growth rate increases mainly because transporter saturation increases, such that more intracellular metabolite (we will call this pyruvate from now on) becomes available. At first, both mixed-acid and homolactic fermentation proteins become saturated, although saturation of homolactic fermentation goes faster because of the different kinetic properties. Since this makes homolactic fermentation more favourable, this induces a switch from mixed-acid to homolactic fermentation. Then, it becomes important that product inhibition by ATP is less for homolactic fermentation. The higher the fraction of fermentation flux through homolactic fermentation becomes, the more favourable it becomes to have higher ATP-concentrations. High ATP-concentrations lead to a more saturated cell synthesis reaction. As a consequence of this adapting ATP-concentration, saturation of homolactic fermentation is faster than linear with growth rate, while the saturation of mixed-acid fermentation decreases. During this switch, proteins are re-allocated from mixed-acid fermentation to homolactic fermentation. However, because proteins are re-allocated within these pathways too (from the homolactic acid reaction to the cell synthesis reaction, and from the cell synthesis reaction to mixed acid fermentation), enzyme concentrations can still remain constant.

As in our model of overflow metabolism, we used a membrane and a cytosolic protein pool constraint, but *L. lactis* differs from for example *E. coli* in that it takes up amino acids instead of synthesizing them. We therefore hypothesized that in a glucose-limited chemostat, *L. lactis* would be energy-limited rather than carbon-limited. We implemented this in our model by considering a biomass reaction of which the rate was determined solely by the ATP-concentration and the corresponding enzyme concentration. Both mixed-acid fermentation and homolactic fermentation generate ATP, but the amount of product inhibition by ATP differs.

At low growth rates, the membrane constraint is the most dominant constraint, and *L. lactis* will therefore maximize ATP-production no matter the cytosolic protein costs involved. Therefore, only the mixed-acid fermentation pathway is used. At higher growth rates (and thus increasing saturation of the glucose transporter) the cytosolic protein pool becomes limiting too. To use the biomass-producing proteins as efficiently as possible, the ATP-concentration must rise, inducing strong product inhibition on the mixed-acid fermentation pathway. This causes a switch from mixed-acid fermentation to homolactic fermentation, and resources are re-allocated from one pathway to another. As a consequence, one could expect enzyme concentrations in the homolactic pathway to rise. However, since at the same time resources are re-allocated within the pathways, the enzyme concentrations can remain constant, see 1.

0.1 Source code

Code for running kinetic model of *L. lactis* The Matlab-code used for the kinetic model of *L. lactis* is attached in a compressed folder as a supplement. In the compressed folder, we have also added a text-file with instructions.

References
