# Supplementary material for: The number of active metabolic pathways is bounded by the number of cellular constraints at maximal metabolic rates
Source: PLoS Comput Biol. 2019 Mar 11;15(3):e1006858. doi: 10.1371/journal.pcbi.1006858 (PMC6428345; doi:10.1371/journal.pcbi.1006858)
Supplement: S4 Appendix — In this short text we first explain how we found EFMs that simultaneously take up several carbon sources in a genome-scale model. We then present the method and results of our own experiments on co-consumption. (PDF) [file pcbi.1006858.s004.pdf]

S4 Appendix: Co-consumption of substrates

Daan H. de Groot, Coco van Boxtel, Robert Planqué, Frank J. Bruggeman, Bas Teusink
January 15, 2019

1 Finding EFMs that co-consume carbon sources

We showed that the number of active EFMs in a growth-maximising micro-organism is bounded by the number of active enzymatic constraints. Since the number of these constraints seems to be low in many experiments, we asked if it could be optimal to co-consume multiple carbon sources given this small number of constraints. We therefore investigated if there is an EFM that by itself consumes two or even three carbon sources and found EFMs for all combinations that we have tested.

The Python- and Matlab-program that we used to find these EFMs is attached to the SI. It works by first performing a Flux Balance Analysis on a genome-scale metabolic network. We have used the *E. coli* model: `iECSE_1348` [1]. In this FBA, the exchange reactions that are set reversible are either essential (trace elements, oxygen, ammonium) or the carbon sources of interested. This makes sure that the optimal solution of this FBA will use these carbon sources. Subsequently, the inactive reactions in the optimal solution are deleted from the network. The resulting smaller network is loaded into Matlab, where a package is used [2] to enumerate the EFMs. These EFMs are checked for co-consumption of the carbon sources.

Note that we find only *if* there is an EFM that co-consumes the carbon sources of interest, not how many EFMs exist that do this.

1.1 Source code

Code for finding coconsumption EFMs The Python and Matlab-code used for finding co-consuming EFMs are attached in a compressed folder as a supplement. In the compressed folder, we have also added a text-file with instructions.

2 Coconsumption experiment

2.1 Method

Strain information
All experiments were performed with *E. coli* strain MG1655.
Growth conditions
The medium employed was the N- C- minimal medium from Gutnick et al. [3], which contains (per liter): K_2SO_4 (1 g), K_2HPO_4 (13.5 g), KH_2PO_4 (4.7 g), $MgSO_4{\cdot}7H_2O$ (0.1 g) and NaCl (2.5 g), supplemented with 20 mM NH_4Cl and thiamine (1mg). After adding saturating amounts of either a single carbon substrate or a combination of multiple (see Table 1), the pH was set to 7.1 using KOH.

Table 1: Carbon mixtures that have been used in combination with the N- C- minimal medium.

Glucose 0.4 % (w/v)	Mannose 20 mM	Maltose 20 mM	Succinate 15 mM	Xylose 20 mM	Abbr
x					G
	x				M
		x			L
			x		S
				x	X
		x	x		SL
	x	x			ML
		x		x	XL
			x	x	XS
	x		x		SM
		x	x	x	SLX
	x	x	x		SLM

Cells were seeded from a frozen glycerol stock into 5 ml liquid N-C- minimal medium +glucose and cultured in 30x115 mm conical tubes, shaking with 220 rpm at 37°C. During the subsequent 12 hours they were sequentially diluted into tubes with the desired medium, to achieve exponential growth and removal of undesired carbon. Then, depending on the specific growth rate in each condition, cells of the various cultures were diluted to different densities and 200 μl of each was transferred to a Greiner 96-well, flat bottom plate. The plate was kept shaking at 37°C and densities were measured at 600 nm using a Spectramax 384 plus (Molecular Devices). Cell densities were chosen such that 8 doublings could take place before growth in the plate could be detected. Every condition was represented by 10 micro-wells (i.e. technical replicates) during each experiment, to make sure enough volume was available for sampling. Samples were taken during growth, filtrated and stored at -20°C for further analysis. This experiment was performed in triplo, meaning that three biological replicates were done on separate days.

Carbon substrate uptake measurements

50μl of undiluted samples were analysed on their mannose, maltose, succinate and xylose content using HPLC (Shimadzo, LC-20AT) at a flow rate of 0.5 mL/min. Calibration samples were made for individual- and triple carbon sources in N-C- minimal medium, to determine the concentrations and validate good separation. Compounds were separated on an ROA-Organic Acid H+ column (Phenomenex, Rezex) and detected using refractive index (Shimadzu, RID-10A) and UV-Vis (Shimadzu, SPD-20A).

Acetate concentrations were measured using an enzyme essay described by Smith et al. [4]. Samples were diluted either 10 or 100 times to stay in the linear range of NADH detection. The essay was conducted in a 96-well, flat bottom plate at 37°C and NADH oxidation was measured at 340 nm using a Spectramax 384 plus (Molecular Devices).

2.2 Data analysis

The three plate-reader experiments resulted in two types of data: OD measurements and HPLC (High Performance Liquid Chromatography) analyses of growth medium samples. The OD measurements were taken every 5 minutes during the full growth experiment and in total 104 growth medium samples were taken at different ODs for all conditions.

The OD measurements were analysed using Matlab. Background OD was subtracted and time

windows of at least two hours were selected in which the natural logarithm of the measurements was sufficiently linear: ($R^2 > 0.95$). For these windows the specific growth rate ($\mu = \frac{1}{OD}\frac{\mathrm{d}OD}{\mathrm{d}t}$) was calculated and the maximum is reported below.

The HPLC analyses were normalized using a peak in the chromatogram that corresponded to a constant compound (phosphate) in the medium. Compound concentrations were calculated using a linear calibration curve that was made for all compounds. Since we were interested in the decrease of substrate concentration, rather than in the absolute value of these concentrations, the concentrations were normalized such that the t_0-concentration is equal to the intended initial concentration for that compound, thereby correcting for small pipetting errors.

2.3 Results

The calculated growth rates for all 12 conditions are summarized in 1. Data can be found in file: `SI_growth_rates.txt`. In almost all cases the growth rate increases or remains equal when an extra compound is added: only the combination of mannose and maltose leads to a lower growth rate than on maltose alone. The addition of succinate to the medium always leads to an increase in growth rate.

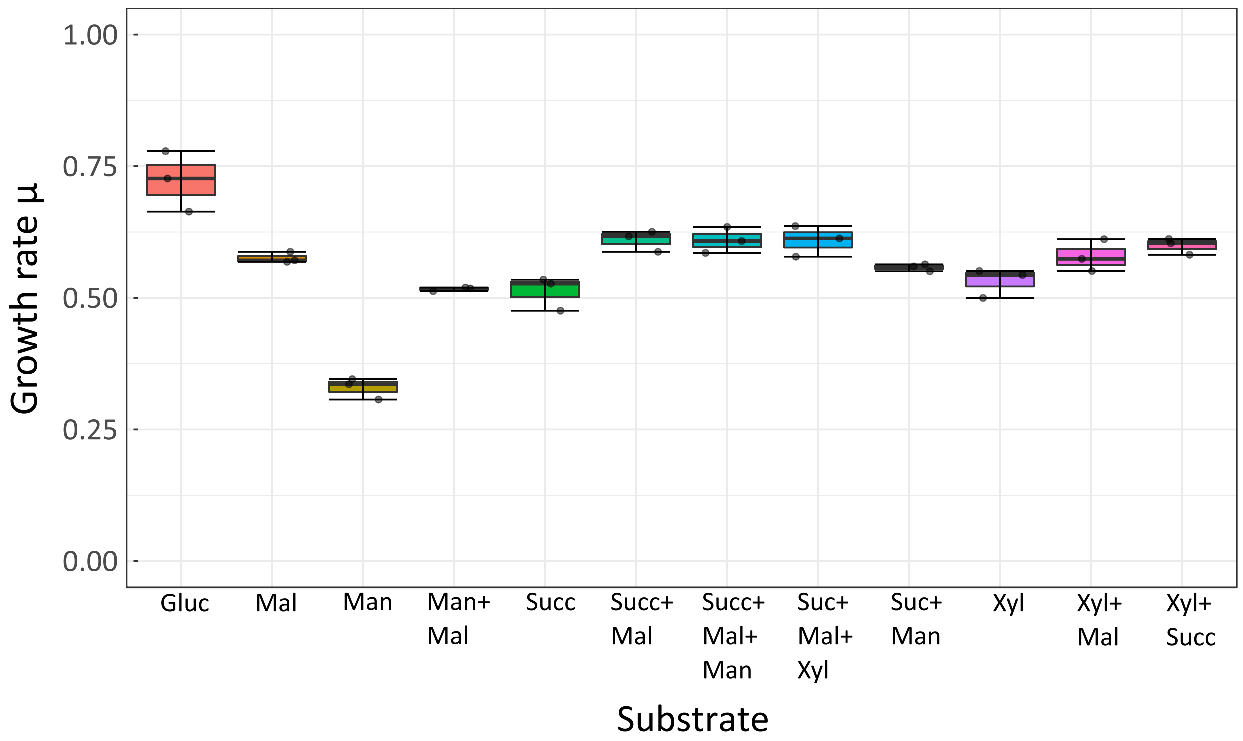

Figure 1: Growth rates were measured for *E. coli* on media with combinations of glucose, maltose, mannose, succinate or xylose. Data from three biological replicates are shown. For each of these replicates, the growth rates from five technical replicates were averaged.

We determined for all conditions the mean specific uptake rate for all the compounds. The resulting data is shown in Table 2 and the standard errors of the means are shown in Table 3. This data can be found in `SI_q_S_comp_cond.xlsx`.

Table 2: Mean specific uptake rate for the 12 conditions and 4 compounds in units: $\frac{\text{mmol}}{\text{OD}_{600}\cdot\text{hour}}$. The letters that indicate the conditions denote the available carbon sources in the medium: S=Succinate, L=maLtose, M=Mannose, X=Xylose, G=Glucose. The uptake rates were estimated from the fitted lines in 2.

	S	SL	M	ML	L	XL	G	XS	X	SLX	SM	SLM
Succinate	80.5	51.0	0.0	0.0	0.0	0.0	0.0	70.0	0.0	46.9	57.0	51.9
Maltose	0.0	59.2	0.0	32.5	71.5	42.0	0.0	0.0	0.0	35.8	0.0	30.2
Xylose	0.0	0.0	0.0	0.0	0.0	31.3	0.0	58.0	64.8	26.9	0.0	0.0
Mannose	0.0	0.0	33.1	27.8	0.0	0.0	0.0	0.0	0.0	0.0	19.0	25.3

Table 3: Standard errors of the mean of specific uptake rate for the 12 conditions and 4 compounds in units: $\frac{\text{mmol}}{\text{OD}_{600}\cdot\text{hour}}$. The letters that indicate the conditions denote the available carbon sources in the medium: S=Succinate, L=maLtose, M=Mannose, X=Xylose, G=Glucose. Standard errors of the mean were calculated from the three biological replicates.

	S	SL	M	ML	L	XL	G	XS	X	SLX	SM	SLM
Succinate	8.6	4.1	0.0	0.0	0.0	0.0	0.0	10.1	0.0	6.6	9.5	7.0
Maltose	0.0	2.1	0.0	1.8	4.0	4.6	0.0	0.0	0.0	2.1	0.0	2.1
Xylose	0.0	0.0	0.0	0.0	0.0	1.8	0.0	2.9	1.9	2.2	0.0	0.0
Mannose	0.0	0.0	1.0	2.4	0.0	0.0	0.0	0.0	0.0	0.0	1.4	3.4

In Figure 2 we plot, for all conditions except for glucose, the relation between the optical density of cells in the sample and the concentrations of the compounds succinate, maltose, mannose and xylose. The dataset can be found in the file SI_OD_conc_per_cond.xlsx.

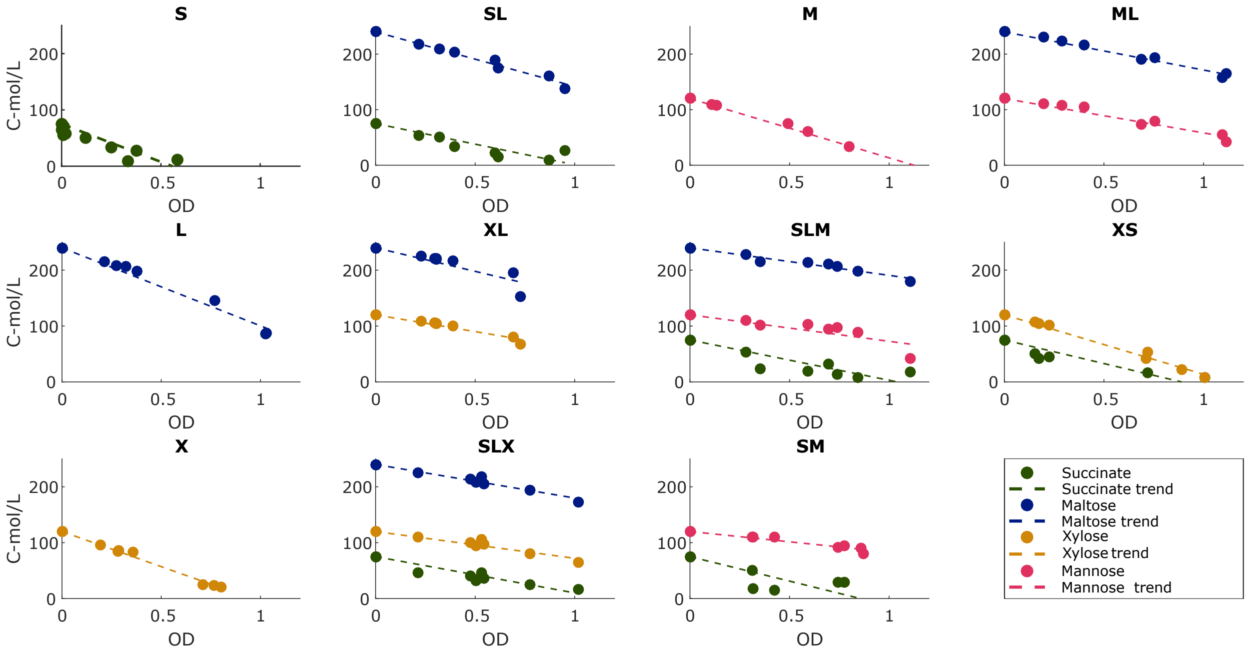

Figure 2: During the growth experiments, the concentration of carbon sources was measured. The letters that indicate the conditions denote the available carbon sources in the medium: S=Succinate, L=maLtose, M=Mannose, X=Xylose, G=Glucose. We here show the decrease in these concentrations as the OD (Optical Density) of the culture increases. Data from three biological replicates was normalized for initial concentration and then shown together. The best linear approximation was calculated and shown by a dashed line.

2.4 Additional datasets and source code for data analysis

Source Code for Data Analysis Coconsumption Experiment All raw data and the Matlab-code used for data analysis can be found in the compressed folder attached to the supplements.

Dataset 1. `SI_growth_rates.txt` Estimated growth rates from separate biological replicates.

Dataset 2. `SI_OD_conc_per_cond.xlsx` For all different growth media, we include an excell-sheet. Shown are the measured concentrations of carbon sources (normalized for initial concentration), with the corresponding Optical Density (OD). The letters that indicate the conditions denote the available carbon sources in the medium: S=Succinate, L=maLtose, M=Mannose, X=Xylose, G=Glucose.

Dataset 3. `SI_q_S_comp_cond.xlsx` Shown are the estimated uptake rates (mean and standard deviation) of different carbon sources (normalized for initial concentration) on the different growth media. The letters that indicate the conditions denote the available carbon sources in the medium: S=Succinate, L=maLtose, M=Mannose, X=Xylose, G=Glucose.

References

[1] Monk JM, Charusanti P, Aziz RK, Lerman JA, Premyodhin N, Orth JD, et al. Genome-scale metabolic reconstructions of multiple *Escherichia coli* strains highlight strain-specific adaptations to nutritional environments. Proceedings of the National Academy of Sciences of the United States of America. 2013;110(50):20338–20343.

[2] Terzer M, Stelling J. Large-scale computation of elementary flux modes with bit pattern trees. Bioinformatics. 2008;24(19):2229–2235.

[3] Gutnick D, Calvo JM, Klopotowski T, Ames BN. Compounds which serve as the sole source of carbon or nitrogen for Salmonella typhimurium LT-2. Journal of Bacteriology. 1969;100(1):215–219.

[4] Smith RF, Humphreys S, Hockaday TDR. The measurement of plasma acetate by a manual or automated technique in diabetic and non-diabetic subjects. Annals of Clinical Biochemistry. 1986;23(3):285–291.
